# Etiology of lower respiratory tract in pneumonia based on metagenomic next-generation sequencing: a retrospective study

**DOI:** 10.3389/fcimb.2023.1291980

**Published:** 2024-01-09

**Authors:** Jin-zhu Wang, Ding Yuan, Xiang-hong Yang, Chang-hua Sun, Lin-lin Hou, Yan Zhang, Yan-xia Gao

**Affiliations:** ^1^ Emergency Department, The First Affiliated Hospital of Zhengzhou University, Zhengzhou, Henan, China; ^2^ Emergency and Critical Care Center, Intensive Care Unit, Zhejiang Provincial People's Hospital (Affiliated People's Hospital), Hangzhou Medical College, Hangzhou, Zhejiang, China

**Keywords:** pulmonary infection, antibiotics, etiology, metagenomic next-generation sequencing, lower respiratory tract

## Abstract

**Introduction:**

Pneumonia are the leading cause of death worldwide, and antibiotic treatment remains fundamental. However, conventional sputum smears or cultures are still inefficient for obtaining pathogenic microorganisms.Metagenomic next-generation sequencing (mNGS) has shown great value in nucleic acid detection, however, the NGS results for lower respiratory tract microorganisms are still poorly studied.

**Methods:**

This study dealt with investigating the efficacy of mNGS in detecting pathogens in the lower respiratory tract of patients with pulmonary infections. A total of 112 patients admitted at the First Affiliated Hospital of Zhengzhou University between April 30, 2018, and June 30, 2020, were enrolled in this retrospective study. The bronchoalveolar lavage fluid (BALF) was obtained from lower respiratory tract from each patient. Routine methods (bacterial smear and culture) and mNGS were employed for the identification of pathogenic microorganisms in BALF.

**Results:**

The average patient age was 53.0 years, with 94.6% (106/112) obtaining pathogenic microorganism results. The total mNGS detection rate of pathogenic microorganisms significantly surpassed conventional methods (93.7% vs. 32.1%, P < 0.05). Notably, 75% of patients (84/112) were found to have bacteria by mNGS, but only 28.6% (32/112) were found to have bacteria by conventional approaches. The most commonly detected bacteria included *Acinetobacter baumannii* (19.6%), *Klebsiella pneumoniae* (17.9%), *Pseudomonas aeruginosa* (14.3%), *Staphylococcus faecium* (12.5%), *Enterococcus faecium* (12.5%), and *Haemophilus parainfluenzae* (11.6%). In 29.5% (33/112) of patients, fungi were identified using mNGS, including 23 cases of *Candida albicans* (20.5%), 18 of *Pneumocystis carinii* (16.1%), and 10 of *Aspergillus* (8.9%). However, only 7.1 % (8/112) of individuals were found to have fungi when conventional procedures were used. The mNGS detection rate of viruses was significantly higher than the conventional method rate (43.8% vs. 0.9%, P < 0.05). The most commonly detected viruses included Epstein–Barr virus (15.2%), cytomegalovirus (13.4%), circovirus (8.9%), human coronavirus (4.5%), and rhinovirus (4.5%). Only 29.4% (33/112) of patients were positive, whereas 5.4% (6/112) of patients were negative for both detection methods as shown by Kappa analysis, indicating poor consistency between the two methods (P = 0.340; Kappa analysis).

**Conclusion:**

Significant benefits of mNGS have been shown in the detection of pathogenic microorganisms in patients with pulmonary infection. For those with suboptimal therapeutic responses, mNGS can provide an etiological basis, aiding in precise anti-infective treatment.

## Introduction

Pulmonary infection stands as a major global cause of mortality, with antibiotic treatment remaining its cornerstone (Magill et al., 2014). In recent times, there has been a shift toward precise anti-infective therapy, emphasizing the early acquisition of an etiological basis to enable the selection of appropriate antibiotics, enhance therapeutic outcomes, and mitigate antibiotic abuse and drug resistance in bacteria (Caliendo et al., 2013). It has been proven that conventional approaches for detecting pathogenic microorganisms, including bacterial smears of sputum or bronchoalveolar lavage fluid (BALF), culture, or nucleic acid detection of pharyngeal swabs, have only a limited level of effectiveness. Numerous studies indicate that these approaches are only capable of identifying pathogenic microorganisms in between 30 and 40% of patients, with even lower detection rates in those who have previously used antibiotics, posing a challenge to clinical requirements (Jain et al., 2015; Torres et al., 2019).

Metagenomic next-generation sequencing (mNGS), as an advanced molecular method of disease diagnosis, provides a high throughput and sensitivity while also being less impacted by exposure to antibiotics. All microbial fragments in a sample may be directly identified by mNGS (Gu et al., 2019; McCombie et al., 2019; Duan et al., 2021). Recently, mNGS has found increased application in clinical settings, proving valuable both in non-infectious diseases (Kamps et al., 2017; Mellis et al., 2018; Cainap et al., 2021) and infectious diseases, particularly in detecting unique infections and emerging pathogens (Afshinnekoo et al., 2017; Simner et al., 2018; Ai et al., 2020; Chen et al., 2020). Studies indicate that the diagnostic performance of mNGS for infectious diseases is 50.7% in sensitivity and 85.7% in specificity (Miao et al., 2018). Despite this, there is still a paucity of mNGS data concerning pathogenic microorganisms in the lower respiratory tract of patients suffering from pulmonary infections, particularly in BALF obtained through bronchoscopy. The focus of this study is to examine mNGS’s efficacy in detecting pathogens in the lower respiratory tracts of individuals with pulmonary infections, in comparison with conventional detection techniques. It seeks to delineate the characteristics of the pathogen spectrum, providing novel insights into precise anti-infective treatment.

## Methods

### Study participants

Patients admitted due to pulmonary infections between April 30, 2018, and June 30, 2020, at the First Affiliated Hospital of Zhengzhou University were included retrospectively. The inclusion criteria were as follows: (1) definite diagnosis according to the criteria outlined in the “*Diagnosis and Treatment of Adults With Community-acquired Pneumonia 2016*” (Metlay et al., 2019) and “*Management of Adults With Hospital-acquired and Ventilator-associated Pneumonia:2016 Clinical Practice Guidelines by the Infectious Diseases Society of America and the American Thoracic Society*” (Kalil et al., 2016) and (2) During the patient’s stay in the hospital, BALF bacterial smear, culture, and mNGS (BALF) to identify pathogenic microorganisms in the respiratory tract. The exclusion criterion comprised complications with other infections and incomplete clinical data. Low immune function was identified as occurring when at least one of the following conditions was present: hematopathy, use of chemotherapeutic drugs and glucocorticoids for autoimmune disorders, immunosuppressant use for solid organ transplantation, neutropenia or chemotherapy for solid tumors in the past 3 months, and impaired immunity owing to other factors, including hereditary or congenital factors.

### Microbiological testing and pathogenic analysis

The bronchoalveolar lavage was performed to obtain BALF. Pathogenic microorganisms were identified using the routine methods of bacterial smear and culture. Simultaneously, mNGS was employed to identify pathogenic microorganisms in BALF. mNGS testing involved immediate storage of 5mL BALF at 4°C, with submission for inspection within two hours. The mNGS procedure for BALF samples includes nucleic acid extraction, library construction, sequencing, and information analysis. mNGS testing is completed by two third party companies(BGI-Huada Genomics Institute, Shenzhen, China;Capital Bio Technology, Beijing, China).

### Acquisition of clinical data and antibiotic treatments

Medical records were queried for clinical data, which included demographic information, lab test outcomes, intensive care unit (ICU) special treatment records, and antibiotic treatment particulars. The following information was also obtained: antibiotic treatment before ICU admission, initial antibiotics administered upon ICU admission, and adjustments made based on mNGS pathogen data.

### Statistical analysis

Data were analyzed statistically utilizing the SPSS 22.0 software. The t-test was used to examine measurement data that conformed to a normal distribution, which were presented as mean ± standard deviation (x ± s). Measurement data that did not conform to a normal distribution were presented as a median (M) and an interquartile range (IQR), with the Mann-Whitney rank sum test applied for comparison. Categorical data were presented as the number of cases and percentages [n (%)], with comparisons conducted using the *χ^2^* test or Fisher’s exact test. Statistical significance was set at *P* < 0.05.

## Results

### Participants’ general characteristics

**Figure 1:**
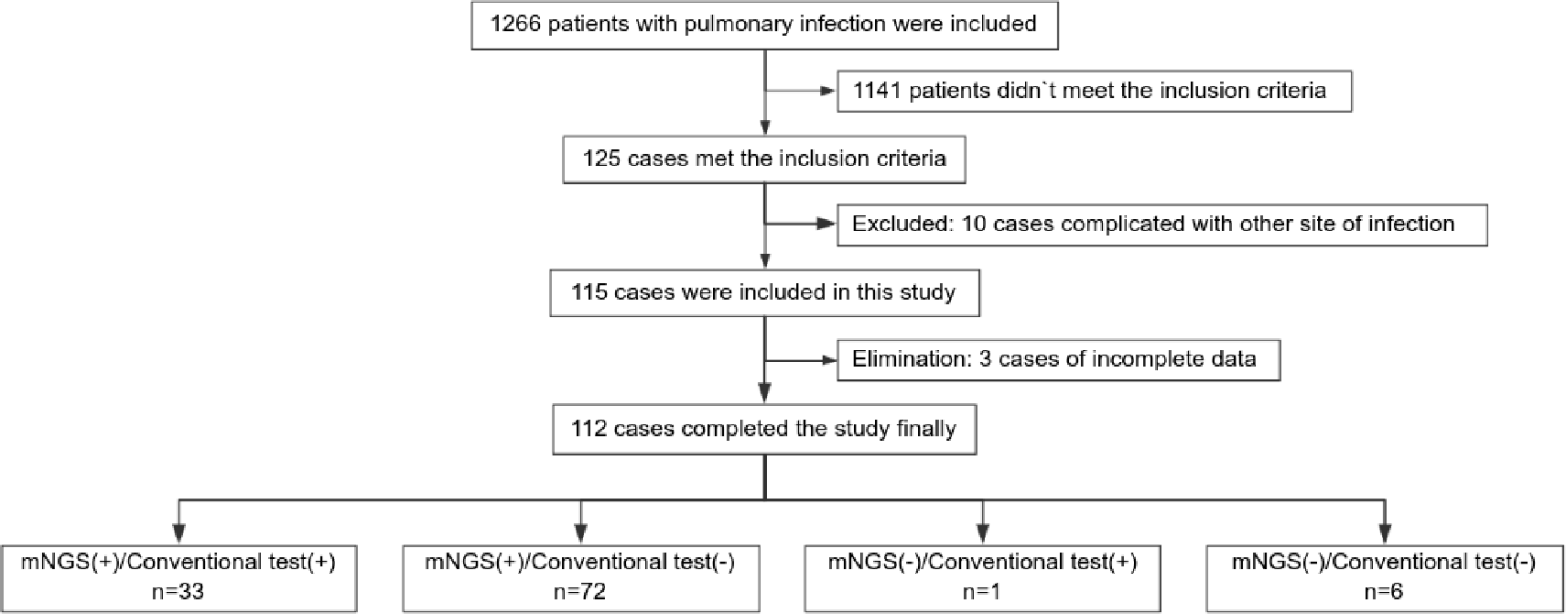
Study flow diagram.

A total of 112 individuals successfully completed the study out of 1,266 patients who received treatment for pulmonary infection between April 30, 2018, and June 30, 2020. Of the total number of participants, 34.0% (34/112) were diagnosed with pulmonary infection for the first time. The mean age of the patients was 53.0 years. Coughing was the most common respiratory symptom, affecting 58.0% of patients (66/112), followed by chest tightness, which affected 39.3% (44/112) of patients. A significant proportion (41.1%) of patients exhibited low immune function. Additionally, 9.8% (11/112) of patients had used glucocorticoids, and 41.1% (46/112) had used antibiotics before admission Furthermore, 39.3% of patients adjusted their antibiotic prescriptions based on pathogenic microorganism results, 4.5% received extracorporeal membrane oxygenation treatment, 59.8% of those patients were admitted to the ICU, and the 30-day hospital mortality rate was 24.1% (**Figure 1**; **Table 1**).

**Table 1:** Clinical and demographic features of 112 pneumonia patients.

Patient characteristics	All patients (n = 112)
Age, years (M, IQR)	53.0 (26.5, 66.0)
Male (n, %)	63 (56.3%)
First visit (n, %)	34 (30.4)
Disease-onset to sampling time, d (M, IQR)	6.0 (4.0, 12.0)
Regional source (n, %)	
Urban area	26 (23.2)
Rural area	86 (76.8)
Smoking (n, %)	22 (19.6)
Alcohol (n, %)	6 (5.4)
Comorbidity (n, %)	
Hypertension	25 (22.3)
Diabetes	11 (9.8)
Heart disease	9 (8.0)
Immune system disease	12 (10.7)
Solid organ transplantation	5 (4.5)
CKD	10 (8.9)
Hematological disease	15 (13.4)
Tumor	6 (5.4)
Immune function deficiency (n, %)	46 (41.1)
Prior use of hormones and antibiotics before admission (n, %)	
Glucocorticoid	11 (9.8)
Fluoroquinolones	10 (8.9)
β-lactam	22 (19.6)
Carbapenem	10 (8.9)
Antiviral medications	3 (2.7)
None	66 (58.9)
T, °C (M, IQR)	37.9 (36.2, 38.8)
HR, bpm (M, IQR)	92.0 (80.5, 106.0)
RR, bpm (M, IQR)	21.0 (19.3, 25.0)
MAP, mmHg (M, IQR)	89.0 (80.0, 95.0)
Inflammation biomarker	
WBC, ×10^9^/L (M, IQR)	9.0 (5.7, 13.0)
NE%, % (M, IQR)	82.9 (70.5, 90.6)
CRP, mg/L (M, IQR)	56.0 (12.1, 124.8)
PCT, ng/mL (M, IQR)	0.20 (0.07, 0.47)
ESR, mm/h (M, IQR)	34.0 (16.0, 68.5)
HGB, g/L (x ± s)	106.3 ± 22.8
PLT, ×10^9^/L (M, IQR)	196.0 (100.5, 307.8)
ICU admission (n, %)	67 (59.8)
Change in antibiotics (n, %)	44 (39.3)
ECMO treatment (n, %)	5 (4.5)
Total 30-day mortality (n, %)	27 (24.1)

M, median; IQR, interquartile range; CKD, chronic kidney disease; T, temperature; HR, heart rate; RR, respiratory frequency; MAP, mean arterial pressure; WBC, white blood cell count; NE, neutrophil; CRP, C-reactive protein; PCT, procalcitonin; HGB, hemoglobin; PLT, platelet; ICU, intensive care unit; ECMO, extracorporeal membrane oxygenation.

### Difference between conventional tests and mNGS in detecting pathogenic microorganisms

**Figure 2:**
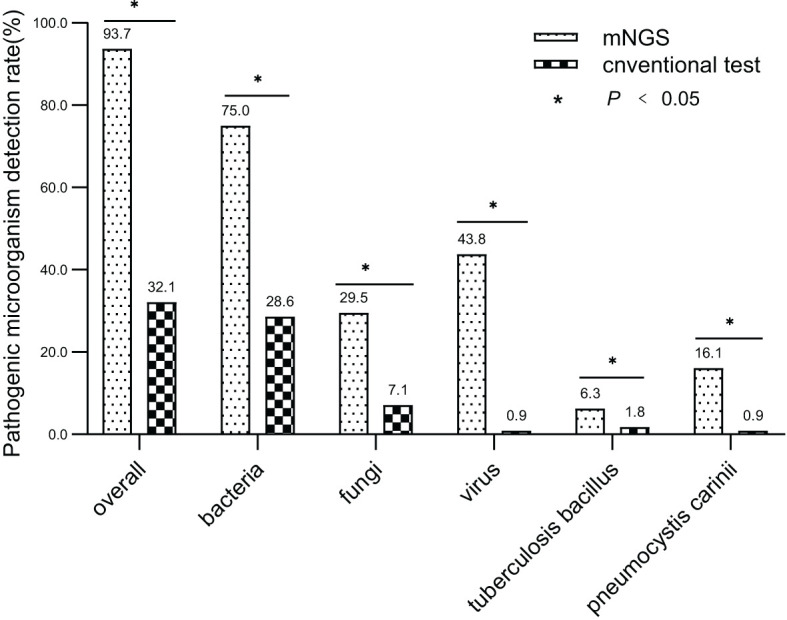
Comparing mNGS-based pathogen detection with conventional detection techniques. (* *P*<0.05).

**Figure 3:**
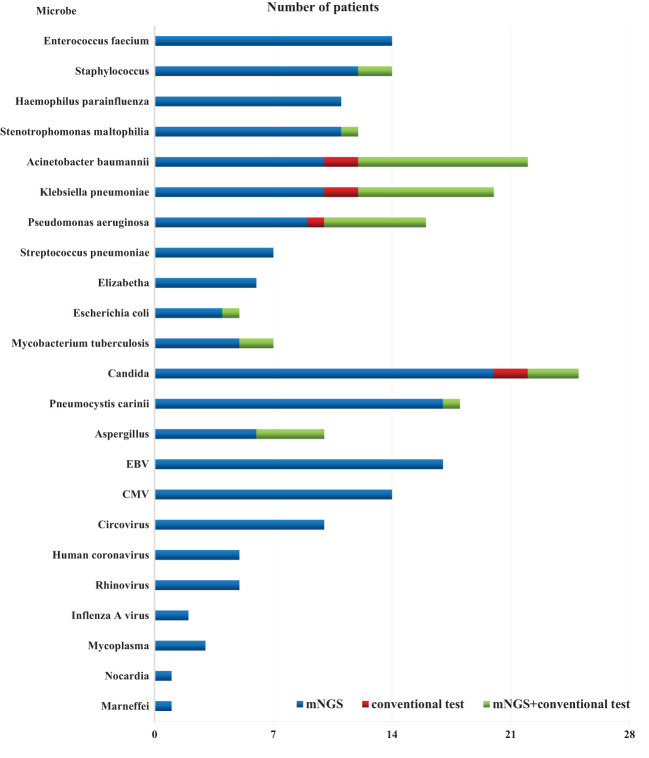
Identification of pathogens by use of mNGS as compared to conventional approaches.

The overall detection rate of mNGS for pathogenic microorganisms demonstrated a significant superiority over conventional methods(93.7% vs. 32.1%, *P* < 0.05) (**Figures 2**, **3**). mNGS identified bacteria in 75.0% (84/112) of patients, whereas conventional methods detected bacteria in only 28.6% (32/112) of patients. Predominantly identified bacteria included *Acinetobacter baumannii* (19.6%), *Klebsiella pneumoniae* (17.9%), *Pseudomonas aeruginosa* (14.3%), *Enterococcus faecium* (12.5%), *Staphylococcus faecium* (12.5%), and *Haemophilus parainfluenzae* (11.6%). Additionally, mNGS detected fungi in 29.5% (33/112) of patients, encompassing *Candida* (23 patients, 20.5%), *Pneumocystis carinii* (18 patients, 16.1%), and *Aspergillus* (10 patients, 8.9%). In contrast, the conventional method identified fungi in only 7.1% (8/112) of patients. The detection rate of viruses by mNGS significantly surpassed that of conventional methods (43.8% vs. 0.9%).The most commonly detected viruses included Epstein–Barr virus (15.2%), cytomegalovirus (12.5%), circovirus (8.9%), rhinovirus (4.5%), and human coronavirus (4.5%). Notably, mNGS detected two cases of Influenza A virus. It is worth mentioning that rare pathogens *Nocardia* and cyanobacteria *Marneffei* were identified in two patients by mNGS.

### Consistency between mNGS and conventional approaches in detecting pathogenic microorganisms

**Figure 4:**
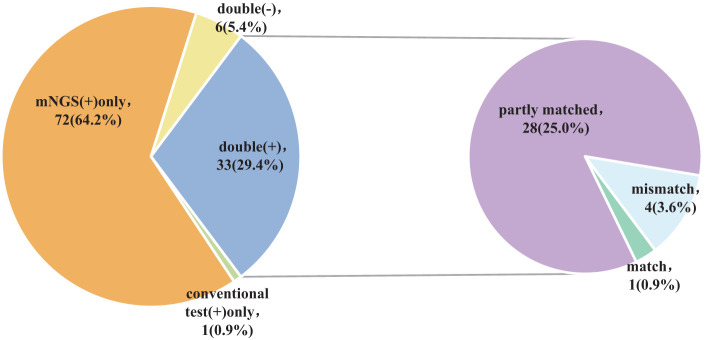
Pathogen identification consistency between mNGS and conventional methods.

The findings of the Kappa analysis suggested that there was poor consistency between the two methods (*P* = 0.340). Notably, both methods yielded positive results in 29.4% (33/112) of cases, while 5.4% (6/112) were negative for both methods. Additionally, 64.2% (72/112) of participants tested positive for mNGS only, and a minimal 0.9% (1/112) showed positivity solely for conventional approaches. Out of the 33 cases who tested positive for both methods, only one (0.9%) exhibited complete matching, whereas 4 cases (3.6%) were entirely mismatched and 28 (25.0%) demonstrated partial matching (**Figure 4**; **Table 2**).

**Table 2:** Consistency analysis of the test outcomes produced by the two methods.

mNGS	Conventional test		Total
	+	−	
+	33	72	105
−	1	6	7
Total	34	78	112

mNGS, metagenomic next-generation sequencing.

Kappa value = 0.030, P = 0.340.

### Comparative analysis of clinical features between patients with positive and negative results detected by routine methods

Among the 105 patients whose mNGS findings were positive, 33 were positive according to the conventional techniques, whereas 72 were negative as per the conventional techniques. Notably, the negative group exhibited a significantly lower proportion of smokers, tumor history, prior use of fluoroquinolones before admission, and admissions to the ICU than the positive group. Additionally, Comparisons between the positive and negative groups showed that patients in the negative group had remarkably lower proportion of neutrophils in peripheral blood(all *P* < 0.05) (**Table 3**).

**Table 3:** Comparative analysis of the clinical features of mNGS (+) patients who obtained positive and negative results outcomes as detected by conventional approaches (n = 105).

Patient characteristics	mNGS (+)/conventional test (+) (n = 33)	mNGS (+)/conventional test (−) (n = 72)	*P* value
Age, years (M, IQR)	55 (41, 68)	50 (25, 65)	0.061
Male (n, %)	24/9	54/18	0.805
First visit (n, %)	10 (27.8)	22 (31.9)	0.979
Disease-onset to sampling time, d (x ± s)	9.0 ± 7.2	8.6 ± 8.8	0.811
Smoking (n, %)	12 (33.3)	9 (13.0)	0.005
Alcohol (n, %)	1 (2.8)	2 (2.9)	0.943
Comorbidity (n, %)			
Hypertension	11 (30.6)	14 (20.3)	0.121
diabetes	6 (16.7)	5 (7.2)	0.081
Heart disease	5 ()	4 ()	0.103
Immune system disease	5 (13.9)	7 (10.1)	0.417
Solid organ transplantation	2 (5.6)	3 (4.3)	0.672
CKD	3 (8.3)	7 (10.1)	0.919
Hematological disease	3 (8.3)	11 (15.9)	0.387
Tumor	5 (13.9)	1 (1.4)	0.005
Prior use of hormones and antibiotics before admission (n, %)			
Glucocorticoid	8 (22.2)	6 (8.7)	0.026
Fluoroquinolones	7 (19.4)	3 (4.3)	0.006
β-lactam	8 (5.6)	10 (10.1)	0.191
Carbapenem	3 (8.3)	5 (7.1)	0.700
Antiviral medications	1 (2.8)	3 (4.3)	0.778
None	13 (36.1)	24 (34.8)	0.546
T, °C (x ± s)	37.1 ± 0.7	37.0 ± 0.8	0.796
HR, bpm (M, IQR)	92.0 (82.0,104.0)	92.0 (80.5,107.7)	0.898
RR, bpm (x ± s)	21.5 ± 3.9	23.4 ± 7.0	0.162
MAP, mmHg (x ± s)	87.7 ± 13.7	88.4 ± 14.3	0.815
Inflammation biomarker			
WBC, ×10^9^/L (x ± s)	11.4 ± 6.4	9.3 ± 5.5	0.083
NE%, % (M, IQR)	88.8 (81.1, 93.8)	79.9 (66.7, 89.6)	0.002
CRP, mg/L (x ± s)	89.8 ± 74.1	67.4 ± 62.4	0.112
PCT, ng/mL (x ± s)	3.6 ± 11.1	2.3 ± 8.7	0.512
ESR, mm/h (x ± s)	55.4 ± 39.3	42.3 ± 34.3	0.088
HGB, g/L (x ± s)	105.9 ± 20.1	105.1 ± 23.6	0.872
PLT, ×10^9^/L (x ± s)	210.3 ± 168.5	225.7 ± 142.9	0.628
ICU admission (n, %)	28 (77.8)	37 (53.6)	0.001
ECMO treatment (n, %)	4 (11.1)	2 (2.9)	0.056
Total 30-day mortality (n, %)	13 (36.1)	14 (20.3)	0.030

mNGS, metagenomic next-generation sequencing; M, median; IQR, interquartile range; CKD, chronic kidney disease; T, temperature; HR, heart rate; RR, respiratory frequency; MAP, mean arterial pressure; WBC, white blood cell count; NE, neutrophil; CRP, C-reactive protein; PCT, procalcitonin; HGB, hemoglobin; PLT, platelet; ICU, intensive care unit; ECMO, extracorporeal membrane oxygenation.

## Discussion

Comparative analysis of mNGS and conventional detection techniques were performed in this retrospective research to detect pathogens in the BALF of patients with pulmonary infection. The findings highlight the exceptional detection capability of mNGS, identifying pathogens even in cases where routine detection methods yielded negative results, offering significant support for antibiotic selection.

In total, 1,266 patients presenting with pulmonary infection were considered for this study, with 112 patients meeting the inclusion criteria (**Figure 1**). Notably, 41.1% (46/112) of these patients had immunodeficiency, 59.8% (67/112) were admitted to the ICU, and 39.3% (44/112) altered their antibiotic regimen based on pathogenic microbiological results (**Table 1**). The study observed that 94.6% of those diagnosed with pulmonary infection obtained pathogenic microorganism results through mNGS or conventional tests (**Figure 2**), aligning with previous research by Wu et al. (Wu et al., 2020). Further analysis revealed that the conventional approaches identified bacteria in only 28.6% of cases, falling significantly short of clinical needs. In contrast, bacteria were detected in 75% of patients using mNGS (**Figure 2**), providing crucial insights for application in clinical settings. The most commonly detected bacteria included *A. baumannii*, *K. pneumoniae*, *P. aeruginosa*, *S. faecium*, *E. faecium*, and *H. parainfluenzae* (**Figure 3**), conforming to typical bacteria prevalent in both hospital- and community-acquired pneumonia (Huang et al., 2020; Wu et al., 2020). With its superior microbial detection rate, mNGS shows great promise for directing antibiotic selection, improving the detection of pathogenic microorganisms, and monitoring the lower respiratory tract in patients who do not respond well to empirical therapy (Chen et al., 2021).

Fungal infections are significant contributors to pneumonia, particularly in patients with risk factors such as immune deficiency, immunosuppressant use, diabetes, organ transplantation, chemotherapy or hormonal therapy, antibiotic misuse, and others (Kauffman, 2015). The study revealed that in comparison to conventional approaches, which only had a 7.1% success rate in detecting fungus, the mNGS detection rate was 29.5% (33/112) (**Figure 2**). Notably, mNGS significantly outperformed conventional approaches in detecting *Pneumocystis carinii* and *Aspergillus*. Given the challenges in detecting fungi, especially with the need for advanced wall-breaking technology and the potential for false negatives, clinicians should interpret results cautiously and consider repeated testing if necessary (Sanmiguel, 2011; Biswas et al., 2017). The study emphasizes the high incidence of fungal infection in patients with immunosuppression, reaching 41.1% (46/112). Conventional methods often face challenges in obtaining an etiological basis for fungal infections in these patients, leading to difficulties in follow-up antifungal treatment. Lack of etiological basis may result in premature discontinuation of antifungal treatment and subsequent recurrence of infection. mNGS proves to be a valuable tool in addressing these challenges. The study reveals that the detection rate of fungi is dramatically increased by mNGS, going from 7.1% (8/112) to 29.5% (33/112), providing clinicians with more references for accurate diagnosis and treatment. Nevertheless, this highlights the need for thorough assessment by clinicians, taking into consideration clinical symptoms, laboratory indicators, imaging results, and the possibility of normal pulmonary fungal colonization, particularly for pulmonary Candida (Zeng et al., 2019). The study emphasizes the importance of considering fungal infections when managing aggravated infections in patients with immunosuppression.

Virus infection indeed plays a crucial role in respiratory tract infections, and conventional methods struggle with low detection rates, even with gene amplification like PCR (Capobianchi et al., 2013; Parker and Chen, 2017). This study underscores that the conventional method’s virus detection rate is a mere 0.9%, while mNGS achieves an impressive 43.8% (**Figure 2**). The most prevalent viruses identified are still Epstein–Barr virus and cytomegalovirus, followed by parvovirus, rhinovirus, and human coronavirus, in agreement with extensive research findings (Chen et al., 2021). Detecting viruses in BALF holds vital clinical significance, though further investigation is needed to determine its clinical relevance in conjunction with specific epidemiology.

In summary, the use of mNGS results in a significant increase in the detection rate of pathogenic microorganisms in BALF, providing an important point of reference for medical practitioners. When the two approaches are compared, it is found that there is a remarkable lack of consistency in the detection rates of pathogenic microorganisms (Kappa value = 0.030, *P* = 0.340) (**Table 2**). Only 29.4% (33/112) of the patients got positive results at the same time,and of the 33 double positive patients, only 1 case (0.9%) was completely matched, 4 cases (3.6%) were completely mismatched, and 28 cases (25.0%) were partially matched (**Figure 4**). This indicates that mNGS can detect pathogenic microorganisms not identified by conventional methods. However, considering the lung’s non-sterile environment and the presence of numerous microorganisms in healthy individuals, clinicians should apply caution when interpreting mNGS data, as they need to distinguish between colonization and infection to determine the most appropriate antibiotic regimen (Erb-Downward et al., 2011; Dickson et al., 2017; Langelier et al., 2018). As observed by Wang et al. (Wang et al., 2019), 26 out of 55 patients with mixed pulmonary infection tested positive using both mNGS and routine approaches, and only 3 (5.5%) cases exhibited matching results, aligning with the current study’s findings. For rare pathogens, conventional methods often struggle with detection, while mNGS proves effective. In this study, 18 cases of Pneumocystis carinii, 7 cases of Mycobacterium tuberculosis, 1 case of *Nocardia*, and 1 case of cyanobacteria *Marneffei* were detected by mNGS (**Figure 3**). With this knowledge, clinicians may choose appropriate antibiotic treatment plans, particularly when conventional methods encounter obstacles such as low gene load, antibiotic exposures, or deficiencies in routine methods for the detection, particularly culture (Chan et al., 2020). Based on the results of this study, physicians should quickly conduct mNGS to identify the types of pathogenic microorganisms in patients who have not responded well to empirical therapy. According to microbial results, 39.3% (44/112) of patients in this study were able to modify their antibiotic prescription, leading to effective treatment outcomes and precise anti-infective medication. The anti-infective treatment was adjusted depending on microbiological findings when mNGS was used to identify respiratory microbes in 21 patients presenting with severe community-acquired pneumonia and poor immune response, as well as in 23 patients with normal immune function. According to these findings, compared with patients with normal immune function, patients with low immune function received fewer full-coverage anti-infective regimens before obtaining pathogenic microbiological results (14.7% vs. 57.1%, *P* = 0.022) and more adjusted anti-infective regimens after obtaining microbiological results (87.0% vs. 57.1%, *P* = 0.027), with >50% of the patients receiving a downgrade in antibiotics.

The analysis of patients who have varying testing results provides valuable insights into the clinical characteristics affecting the detection of pathogenic microorganisms. Patients who had negative findings in the mNGS test showed a significantly lower proportion of smokers, tumor history, prior use of fluoroquinolones before admission and admissions to the ICU than the positive group, and decreased proportion of neutrophils in peripheral blood (**Table 3**). This shows that clinicians may have a more difficult time finding pathogenic microorganisms in patients who do not smoke, patients with a history of cancer, patients exposured to fluoroquinolones before admission, patients who are treated in general wards, and patients whose inflammatory indices are low. For such patients, the study recommends timely mNGS detection of BALF, especially if the initial anti-infective treatment proves ineffective. The implementation of mNGS in these cases is highlighted as a crucial step to significantly improve the detection rate of pathogenic microorganisms. Critically ill patients in the ICU, with established artificial airways, find it relatively easier to obtain BALF through bedside tracheoscopy. However, patients in general wards with milder illnesses face challenges due to the discomfort caused by tracheoscopy as an invasive procedure (Wu et al., 2020).

At present, new nucleic acid detection techniques have been used to detect pathogenic microorganisms. Droplet digital PCR (ddPCR) is a method of absolute nucleic acid quantification by using Poisson statistical analysis of the number of positive and negative droplets and has been proven to achieve higher accuracy and sensitivity than qPCR (Shirai et al., 2022). It has been used in a lot of clinical fields, such as liquid biopsies for cancer monitoring and noninvasive prenatal testing for genetic abnormality detection(Cainap et al., 2021; Boldrin et al., 2022). However, due to the testing of specimens, it is currently limited to the detection and diagnosis of bloodstream infections (Lin et al., 2023). It has great clinical value in patients with lung infections combined with bloodstream infections, especially because ddPCR detects the same pathogen in the blood as the lower respiratory tract secretions (Wu et al., 2022).

Acknowledging the study’s limitations is crucial. First, the presentation solely focuses on the findings of the examination into the presence of pathogenic microorganisms in the lower respiratory tract, leaving the judgment of whether they are pathogenic or colonized bacteria to clinicians. Second, considering that it was conducted in a single site retrospectively, there is a possibility that it was subjected to selection bias. Prospective and multicenter studies are necessary for further confirmation. Third, mNGS technology has some inherent flaws, with detection results prone to contamination by the kit-related background microorganisms and nucleic acids. Advancements and updates in mNGS technology are expected to address this issue.

## Conclusions

In conclusion, mNGS demonstrates significant advantages in detecting pathogenic microorganisms in the lower respiratory tract of individuals suffering from pulmonary infections, offering valuable references for clinicians. As a novel molecular diagnostic tool, the ongoing innovation in mNGS technology is anticipated to provide even more substantial assistance to clinicians.

## Data availability statement

The original contributions presented in the study are included in the article/supplementary materials. Further inquiries can be directed to the corresponding author.

## Ethics statement

The studies involving humans were approved by Scientific Research and Clinical Trial Ethics Committee of the First Affiliated Hospital of Zhengzhou University (approval number: 2021-KY-0952-003). The studies were conducted in accordance with the local legislation and institutional requirements. The participants provided their written informed consent to participate in this study.

## Author contributions

J-ZW: Writing – original draft. DY: Data curation, Investigation, Writing – review & editing. X-HY: Funding acquisition, Project administration, Writing – review & editing. C-HS: Methodology, Resources, Writing – review & editing. L-LH: Data curation, Resources, Writing – review & editing. YZ: Data curation, Formal analysis, Writing – review & editing. Y-XG: Supervision, Writing – review & editing.
